# Supplementary material for: Directed functional and structural connectivity in a large-scale model for the mouse cortex
Source: Netw Neurosci. 2021 Nov 30;5(4):874–89. doi: 10.1162/netn_a_00206 (PMC8746117; doi:10.1162/netn_a_00206)
Supplement: Supplementary file 1 [file netn-05-874-s001.pdf]

Nunes, R. V., Reyes, M. B., Mejias, J. F. & de Camargo, R. Y. (2021). Supporting information for "Directed functional and structural connectivity in a largescale model for the mouse cortex." *Network Neuroscience*. Advance publication. https://doi.org/10.1162/netn_a_00206

Journal: **NETWORK NEUROSCIENCE**

SUPPLEMENTARY INFORMATION

Directed functional and structural connectivity in a large-scale model for the mouse cortex

Ronaldo V. Nunes[1], Marcelo B. Reyes[1], Jorge F. Mejias[2], and Raphael Y. de Camargo[1]

[1]Center for Mathematics, Computing and Cognition, Universidade Federal do ABC, São Bernardo do Campo, Brazil

[2]Swammerdam Institute for Life Sciences, University of Amsterdam, Amsterdam, The Netherlands

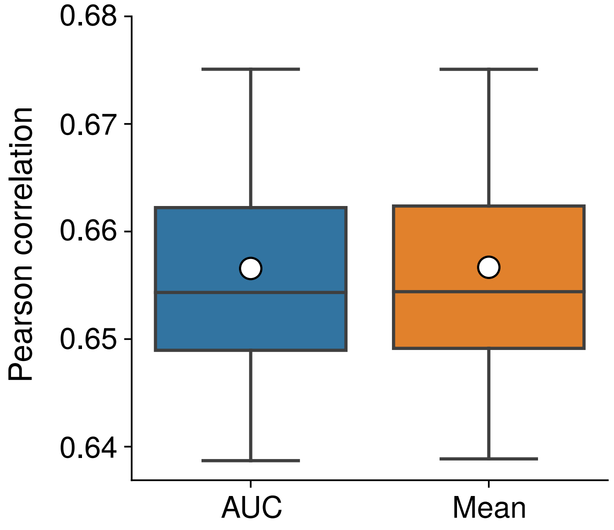

Figure S1. Distribution of correlation between area under curve (AUC) of GPDC estimates or mean of GPDC and FLN. In blue, distribution of Pearson correlations between AUC of GPDC and FLN. The average Pearson correlation is 0.656. In orange, distribution of Pearson correlations between mean of GPDC and FLN. The average Pearson correlation is also 0.656.

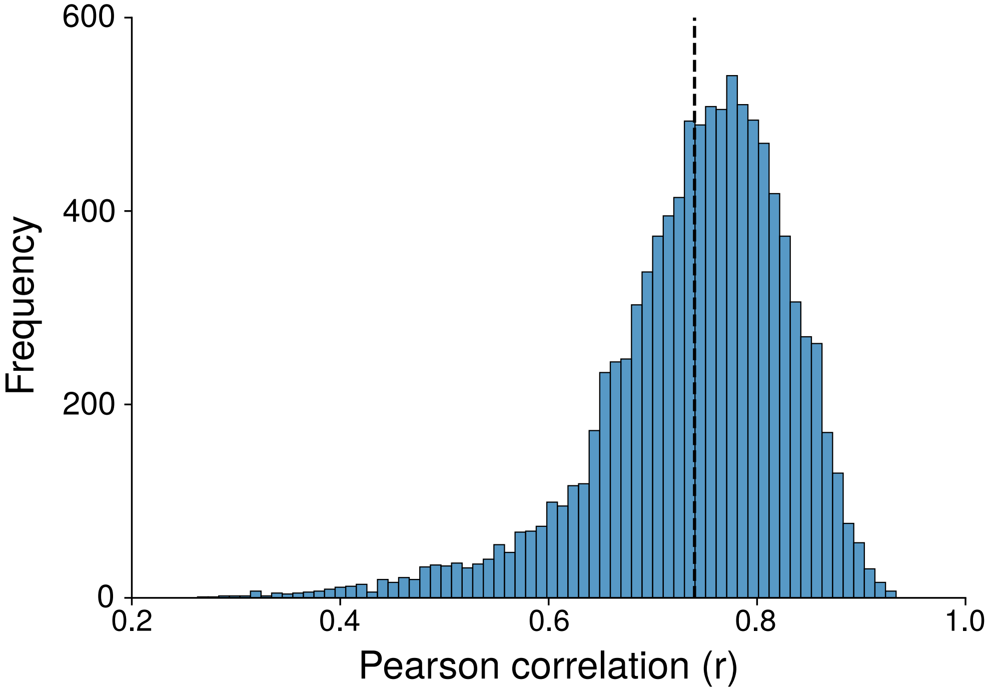

Figure S2. Distribution of correlation between FLN and GPDC for 1000 bootstrap samples of 80 randomly selected edges. In each bootstrap sample, it was computed the correlation for each simulation separately, involving a total of 10000 samples (10 simulation x 1000 bootstrap samples). The dashed line is the mean of the distribution ($\bar{r} = 0.74$).

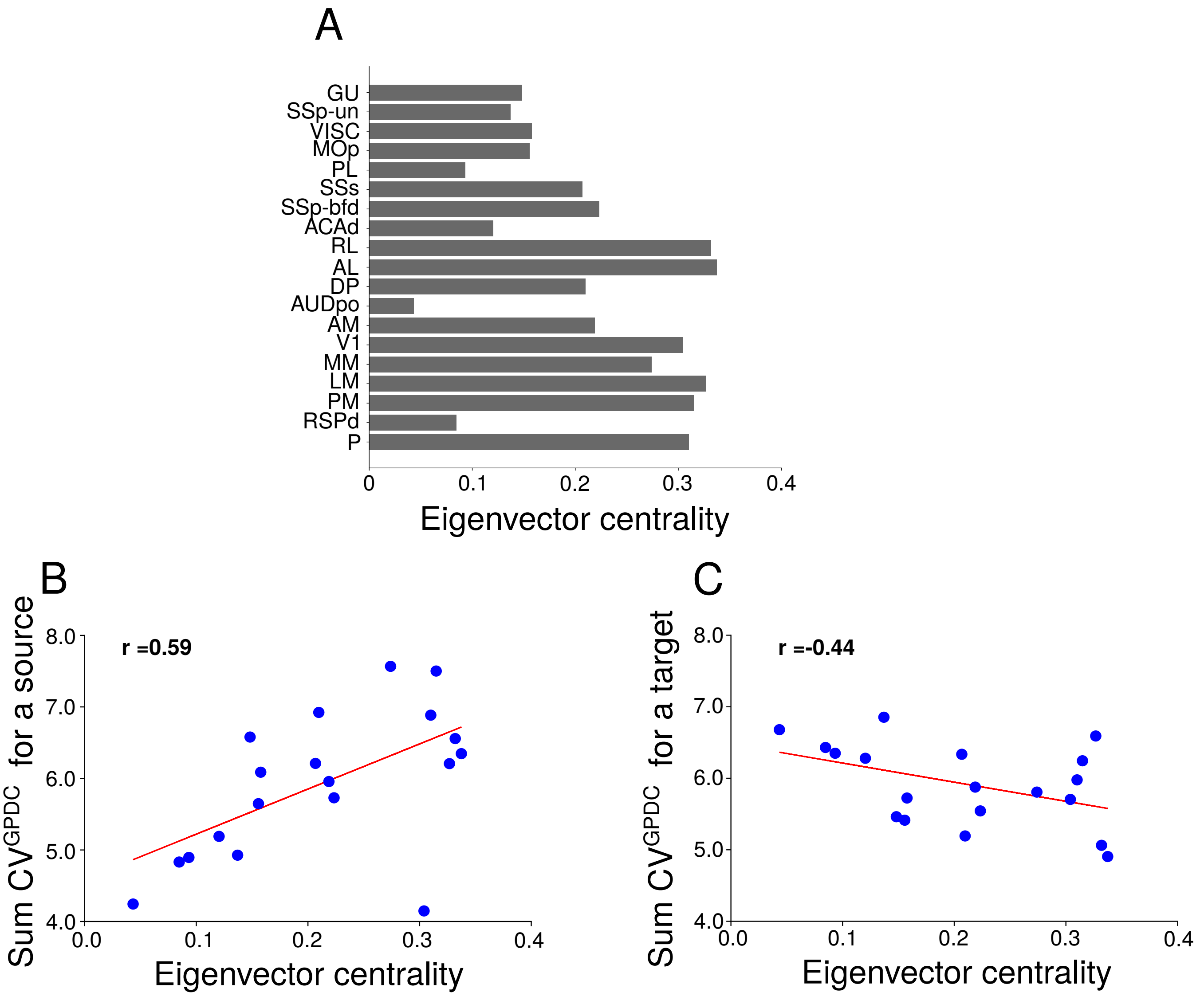

7 **Figure S3. Relationship between eigenvector centrality and varibility of GPDC.**. A) Eigenvector centrality for all cortical areas. B) Sum of $\mathrm{CV}^{\mathrm{GPDC}}$ for
8 a source vs. eigenvector centrality. C) Sum of $\mathrm{CV}^{\mathrm{GPDC}}$ for a target vs. eigenvector centrality.

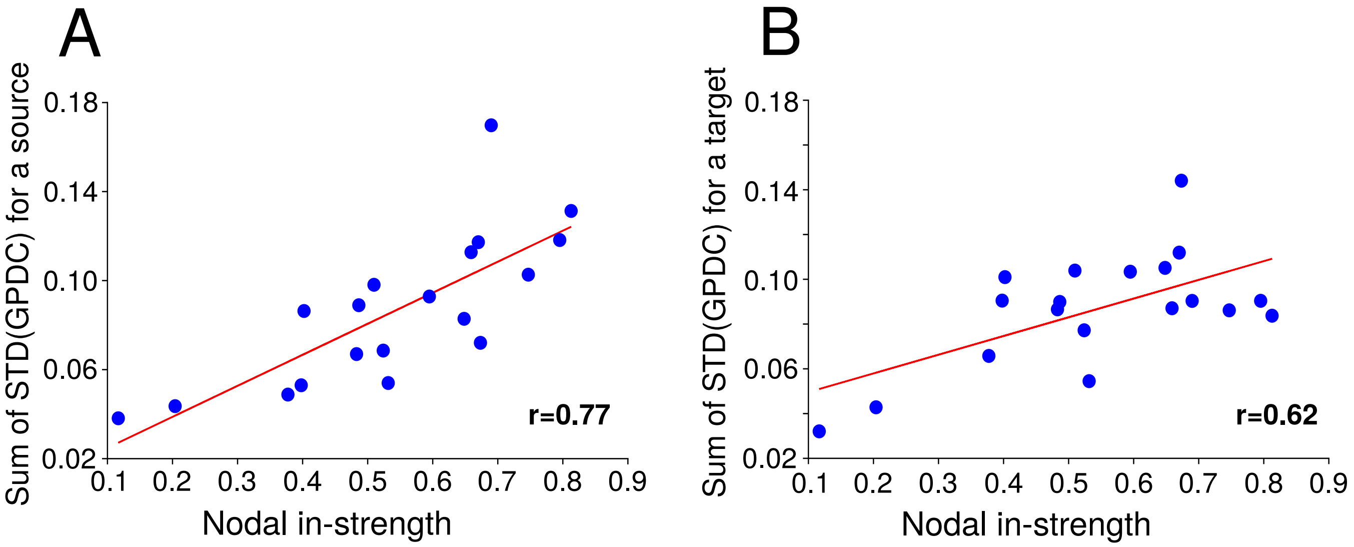

9 **Figure S4. Standard deviation of GPDC and centrality.**. A) Sum of standard deviation of GPDC for a source $vs.$ nodal in-strength. C) Sum of standard
10 deviation of GPDC for a target $vs.$ nodal in-strength.

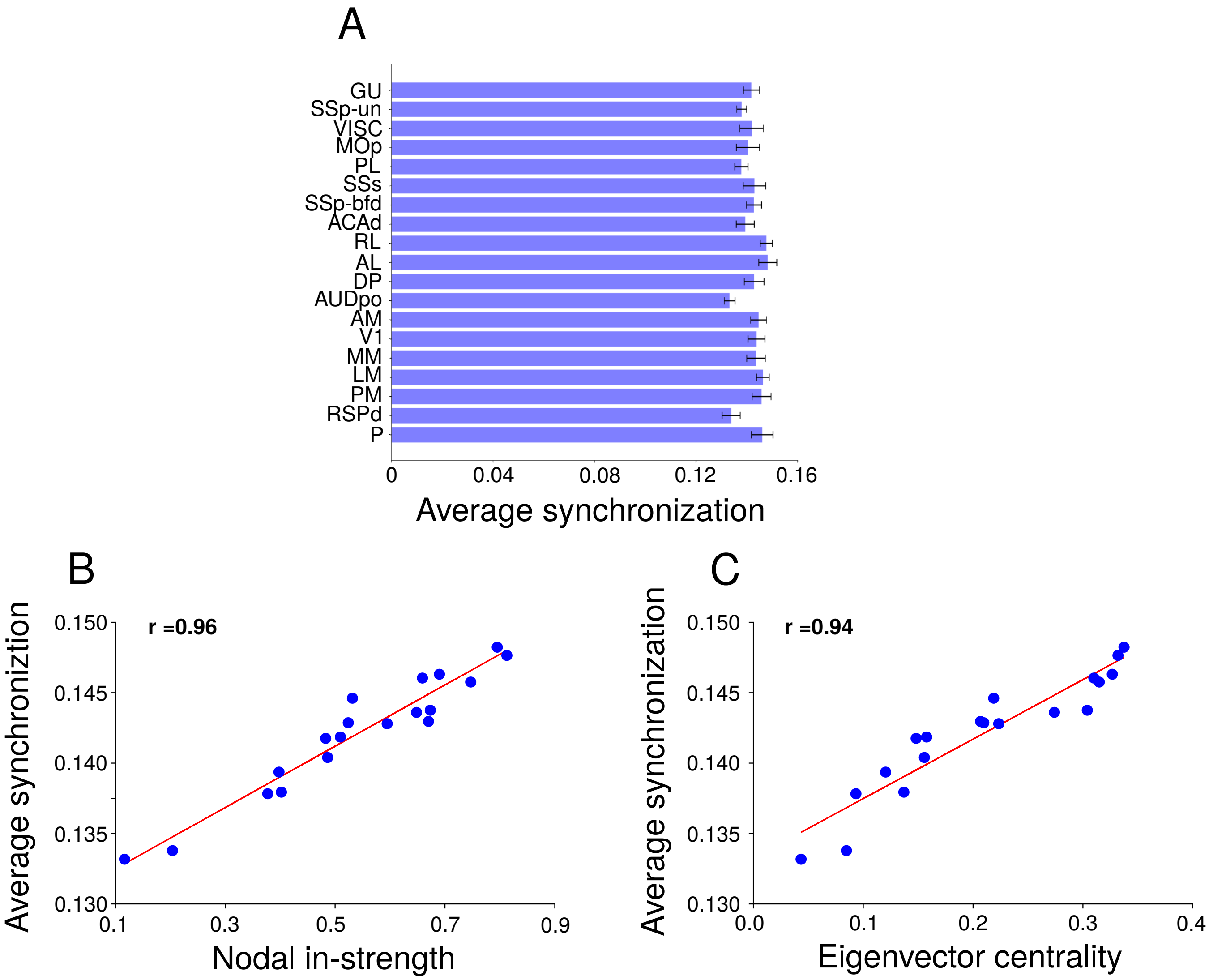

Figure S5. Synchronization $vs.$ **centrality.** A) Average synchronization (average over simulation of average synchronization over time). Bars are standard deviation. B) Average synchronization vs nodal in-strength. C) B) Average synchronization vs eigenvector centrality. Synchronization was obtained using PySpike (Mulansky & Kreuz, 2016). Eigenvector centrality was computed using NetworkX (Schult & Swart, 2008).

Table S1. Name of areas in mouse cortical connectome. Adapted from (Gămănuţ et al., 2018)

**Abbreviations**	**Areas**
ACAd	Anterior cingulate area dorsal part
AL	Anterolateral area
AM	Anteromedial area
AUDpo	Auditory cortex posterior area
DP	Dorsal posterior area
GU	Gustatory area
LM	Lateromedial area
MM	Mediomedial area
MOp	Motor cortex primary
P	Posterior area
PL	Prelimbic area
PM	Posteromedial area
RL	Rostrolateral area
RSPd	Rostroplenial area dorsal part
SSp-bfd	Somatosensory cortex primary barrel field
SSp-un	Somatosensory cortex primary unassigned
SSs	Somatosensory cortex secondary
V1	Primary visual area
VISC	Visceral area

AKAIKE'S INFORMATION CRITERION (AIC)

The AIC for order p is obtained by

$$\mathrm{AIC}(p) = \ln(\det(\Sigma_p)) + \frac{2pN^2}{T}, \tag{S1}$$

Σ_p is the covariance matrix of residuals for the model with order p, N is the number of time-series and T is the length of time-series (Sameshima & Baccala, 2014).

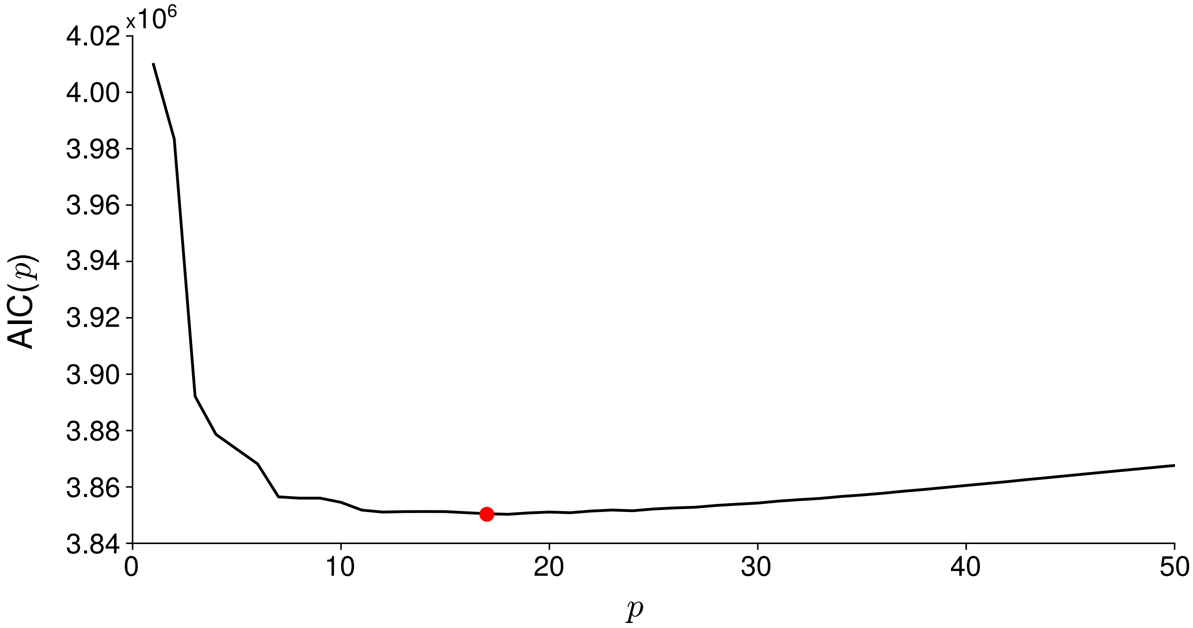

Figure S6. AIC for the analysis of one simulation. The best order (red bullet) is 17 with AIC(17) $\approx$ 3850286. For all simulations analyzed in Figure 2 the best order was 17 where the average AIC(17) over 10 simulations was 3855348.8 and the standard deviation was 4855.3.

Table S2. AIC order p for each cluster size. Minimum, median and maximum of distribution of AIC order p for GPDC computed for each cluster size. The distribution consider GPDC computed for all simulations and all randomly chosen areas.

**Cluster size**	**Minimum p**	**Median p**	**Maximum p**
3	18	21	39
4	18	21	33
5	18	21	24
6	18	21	24
7	18	21	24
8	18	18	24
9	18	18	21
10	18	18	21
11	18	18	21
12	18	18	21
13	18	18	21
14	18	18	21
15	18	18	21

REFERENCES

Gămănuţ, R., Kennedy, H., Toroczkai, Z., Ercsey-Ravasz, M., Van Essen, D. C., Knoblauch, K., & Burkhalter, A. (2018). The mouse cortical connectome, characterized by an ultra-dense cortical graph, maintains specificity by distinct connectivity profiles. *Neuron*, *97*(3), 698–715.

Mulansky, M., & Kreuz, T. (2016). Pyspike—a python library for analyzing spike train synchrony. *SoftwareX*, *5*, 183–189.

Sameshima, K., & Baccala, L. A. (2014). *Methods in brain connectivity inference through multivariate time series analysis*. CRC press.

Schult, D. A., & Swart, P. (2008). Exploring network structure, dynamics, and function using networkx. In *Proceedings of the 7th python in science conferences (scipy 2008)* (Vol. 2008, pp. 11–16).
